# Novel MR imaging method – MAVRIC – for metal artifact suppression after joint replacement in musculoskeletal tumor patients

**DOI:** 10.1186/s12891-015-0838-1

**Published:** 2015-12-04

**Authors:** Michiro Susa, Sota Oguro, Kazutaka Kikuta, Kazumasa Nishimoto, Keisuke Horiuchi, Masahiro Jinzaki, Masaya Nakamura, Morio Matsumoto, Kazuhiro Chiba, Hideo Morioka

**Affiliations:** Department of Orthopaedic Surgery, National Defense Medical College, 3-2 Namiki, Tokorozawa, Saitama 359-8513 Japan; Department of Orthopedic Surgery, Keio University School of Medicine, 35 Shinanomachi, Shinjuku-ku, Tokyo 160-8582 Japan; Department of Diagnostic Radiology, Keio University School of Medicine, 35 Shinanomachi, Shinjuku-ku, Tokyo 160-8582 Japan

**Keywords:** MRI, MAVRIC, Metal artifact

## Abstract

**Background:**

Standard imaging modality for the follow-up after prosthetic replacements for musculoskeletal tumor patients has been conventional radiography. This technique is effective in detecting subtle changes in bone adjacent to metal implants, but in many cases, radiographs do not lead to definitive diagnosis of postoperative adverse events such as acute infection, local recurrence of soft tissue tumor or soft tissue local recurrence of osseous sarcoma. Conventional MRI sequences have not been effective due to metal artifacts. In this study, we tried to elucidate the effectiveness of metal artifact suppression using novel sequence, multiacquisition variable-resonance image combination (MAVRIC), after musculoskeletal tumor surgeries.

**Methods:**

We retrospectively analyzed 5 cases of malignant bone and soft tissue sarcoma patients who were reconstructed with metal prosthesis after wide resection of tumors. Images obtained using MAVRIC and short tau inversion recovery (STIR) were compared side by side. The paired MAVRIC and STIR images were qualitatively compared independently by two specialists for 4 parameters: visualization of bone - implant interface, visualization of surrounding soft tissues, image blurring, and overall image quality. Quantitatively, paired images were reviewed to identify the slice where the metal artifact was maximal, and a region of interest encompassing the implant and surrounding artifact was drawn using Advantage Workstation (GE Healthcare, Japan).

**Results:**

There were no local recurrences that were detected. By utilizing MAVRIC, visualization of the bone - implant interface and visualization of the surrounding soft tissue were significantly improved in MAVRIC compared to STIR. Although blurring was worse on the MAVRIC acquisitions, the overall image quality was still better on MAVRIC. Quantitatively, the area of metal artifact measured using MAVRIC was markedly less compared to STIR (61.4 cm^2^ vs 135.9 cm^2^).

**Conclusion:**

Despite the relatively small number of cases in the present study, our observation strongly suggests that MAVRIC is able to improve the quality of images by decreasing the artifact caused by endoprosthesis, frequently utilized in reconstruction of musculoskeletal tumor patients. Further installments of conventional imaging sequences with the addition of gadolinium - enhancement will enable increased accuracy in diagnosing local recurrences of sarcoma patients.

## Background

Multimodal therapy for sarcoma patients has improved the prognosis in the past decades, but surgery is still the mainstay of treatment for malignant tumors. Surgery for sarcoma of both bone and soft tissue usually consists of wide resection including the cuff of normal tissue, which leads to large defects. When skeletal structure is compromised, especially near the joint, endoprosthesis is frequently utilized for the reconstruction. Traditionally, radiograph was utilized to detect the change in bone, but it has not enabled the detection of devastating postoperative events such as soft tissue recurrences. The usage of massive metal implants have prevented the usage of magnetic resonance imaging (MRI) which has high spatial resolution and tissue contrasts compared to plain radiographs due to metal artifacts.

Recently, improvements in metal composition of prosthesis [1, 2] and artifact-reducing MRI sequences have been reported [3–5]. Multiacquisition variable-resonance image combination (MAVRIC) was first reported in 2009, where it uses multiple excitations to excite the overall volume being imaged [3]. MAVRIC excites a series of limited spectral distortion and uses the three-dimensional (3D) spin-echo acquisition to resolve the profile of each excited slice in the region of interest. MAVRIC has been reported to reduce the metal artifacts around smaller metal implants usually reserved for conventional orthopaedic procedure [4, 6, 7]. In this study, we analyzed the effectiveness of MAVRIC for suppression of metal artifacts around larger tumor endoprosthesis for early possible detection of tumor recurrences.

## Methods

**Fig. 1:**
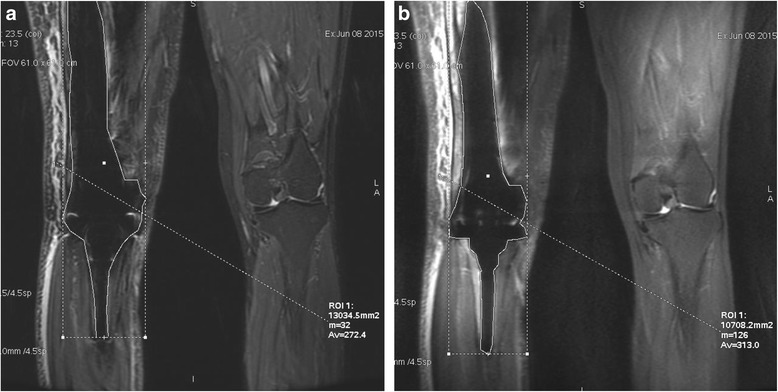
**a**. Conventional STIR sequence obained after distal femoral endoprosthesis reconstruction after chondrosarcoma resection in a 46 year - old female. **b**. MAVRIC image. The maximum area of metal artifact was measured in a matching plane for both images. Area of the artifact was 130.3 cm^2^ for STIR and 107.0 cm^2^ for MAVRIC

Since 2014, 4 megaprosthesis (1 total femur, 1 proximal femur, 1 distal femur, and 1 proximal humerus) and 1 augmented proximal femoral reconstruction stem were utilized for reconstructions and underwent MR imaging on a 3T scanner (Discovery 750, GE Healthcare, Japan). Histological diagnosis included 2 osteosarcomas, 2 chondrosarcomas and 1 synovial sarcoma. There were 4 female and1 male. Proximal humeral implant and reconstruction stem were composed from titanium alloy and all other implants were made up of cobalt-chrome. Conventional short tau inversion recovery (STIR) and MAVRIC sequences were acquired in the coronal planes using similar scan parameters and readout bandwidth. MAVRIC sequences were obtained with a 3T scanner (GE SIGNA discovery MR 750, GE Healthcare, Milwaukee, WI) in the supine position. The protocol included coronal, STIR images (TR/TE: 4500–6700/6.3–6.8 ms; inversion time 175 ms; slice thickness 4.5 mm; gap 0 mm; flip angle 65°; field of view 280 mm, matrix 320 × 320, 24 slices, band width 125 kHz, acquisition time from 4 min 15 s to 6 min 32 s) and coronal proton density weighted images (TR/TE: 2400/6.3–7.1 ms; slice thickness 4.5 mm; gap 0 mm; flip angle 75°; field of view 280 mm, matrix 384 × 320, 24 slices, band width 125 kHz, acquisition time from 4 min 5 s to 6 min 7 s) with an 32 – channel torso surface coil. These MR images were retrospectively analyzed between two musculoskeletal tumor specialists (one radiologist and one orthopaedic oncologist) in a matching anatomic plane with the maximal artifact for both qualitative and quantitative variables. Artifact was defined as areas of signal void pile-up or geometric distortion. Images were graded on a five-point scale from −2 to +2, where lower score suggested better outcome for MAVRIC acquisitions, which was previously reported by Gutierrez et al. [8]. −2 indicate that MAVRIC is significantly better, and −1 meant somewhat better compared to STIR. Readers were blinded as to the type of sequence that they were grading. Quantitatively, plane with the maximal artifact was determined, and region of interest was measured using Advantage Workstation (GE Healthcare, Japan) (Fig. 1). This study was conducted with the approval of Institutional Review Board of Keio University and all participants gave their informed consent to assessing and using their data.

## Results

**Fig. 2:**
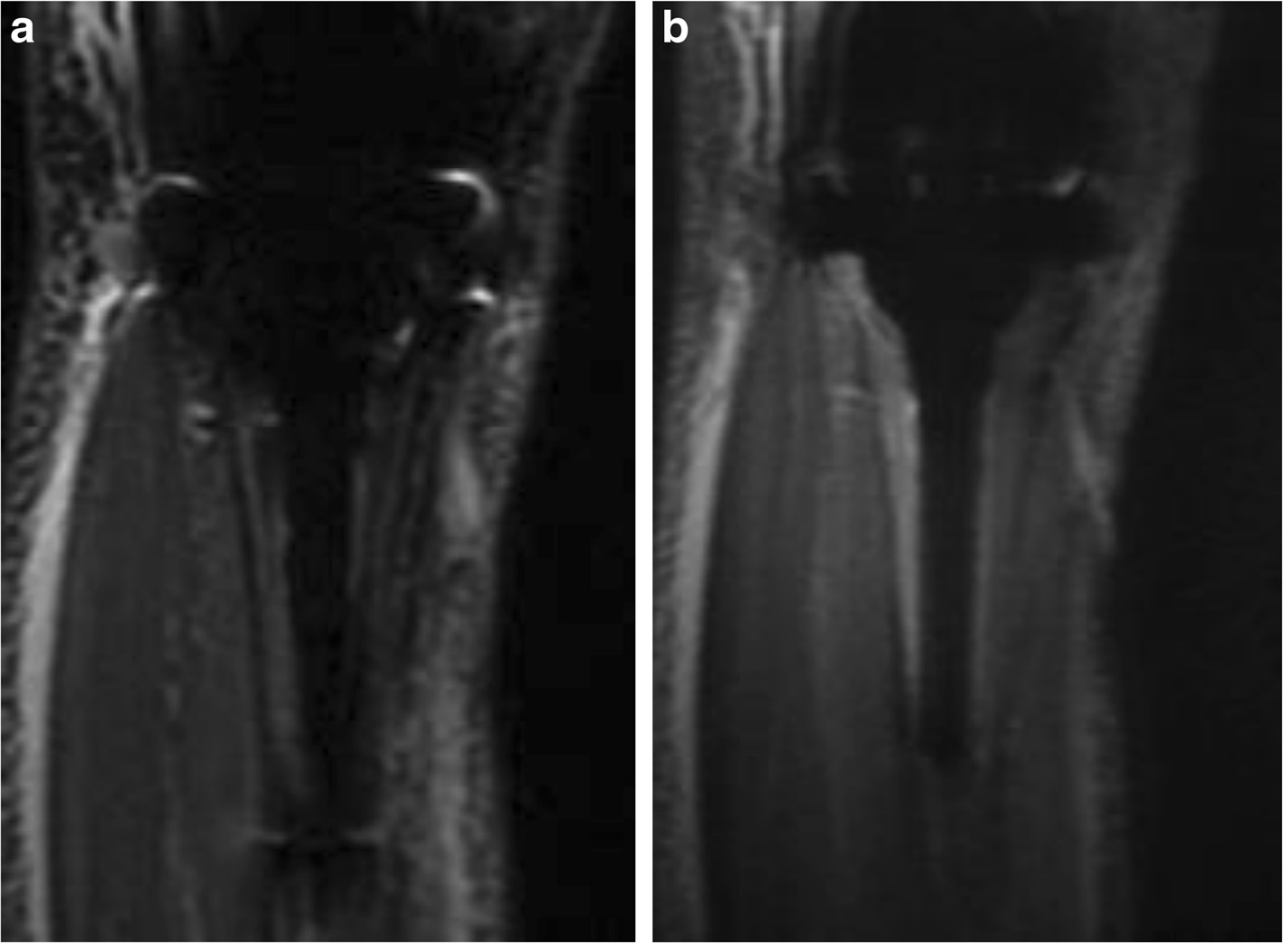
**a**. Conventional STIR image and **b**. MAVRIC image were compared for bone and implant interface. MAVRIC was able to depict the bone immediately adjacent to the endoprosthesis. Additionally, because of the reduced artifact, the prosthesis itself is now visible as signal void especially around the knee

**Fig. 3:**
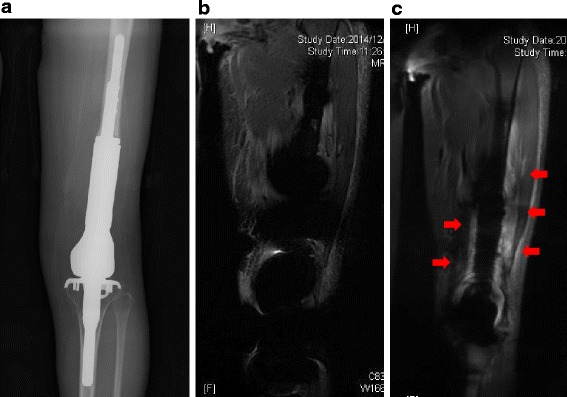
**a**. A 27 year - old female received a distal femoral endoprosthetic reconstruction after wide resection of an osteosarcoma 6 years prior to the exmaination. **b**. Conventional STIR image and **c**. MAVRIC image were compared for the depiction of surrounding soft tissue adjacent to the endoprosthesis. Joint effusion is observed surrounding the metal implant in MAVRIC image (*red arrow*). Due to early detection of the periprosthetic infection, thorough irrigation and debriedement was performed, and the patient is well without recurrence

**Fig. 4:**
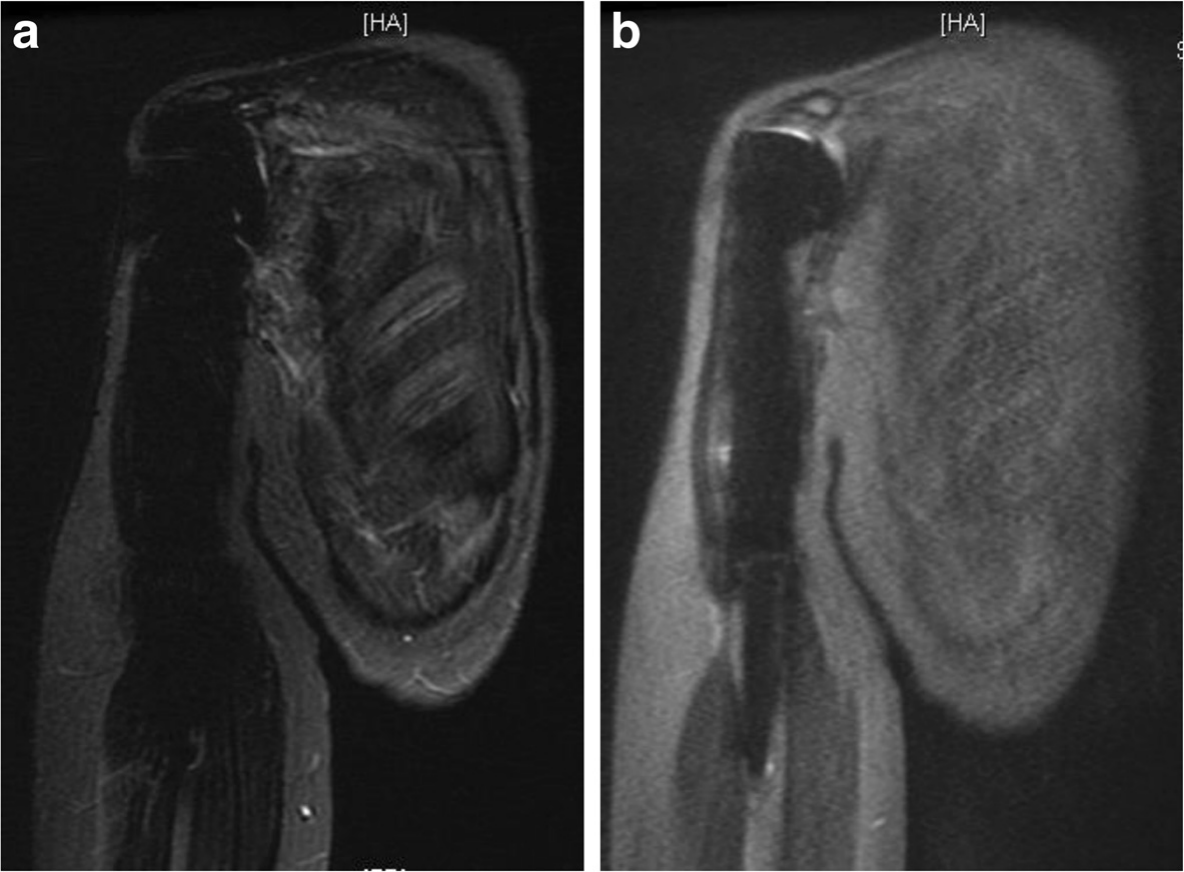
**a**. STIR image of a proximal humeral endoprosthetic reconstruction and **b**. MAVRIC image were compared side by side for the qualitaive assesment of the blurring. Image quality of the MAVRIC is reduced compared to STIR with blurry contours, lower contrast, and lower resolution as depicted by the ribs (average grade: +1.12). The fat saturation is clearly reduced with the MAVRIC as seen in the subcutaneous fat tissue

There were no local recurrences that were detected and no complications such as local hotness during the examination. The quality of images were compared and scored independently between the 2 observers. By utilizing MAVRIC, visualization of the bone - implant interface (average grade: −1.88) (Fig. 2) and visualization of the surrounding soft tissue (average grade: −1.75) (Fig. 3) were significantly improved in MAVRIC compared to STIR. Although blurring was worse on the MAVRIC acquisitions with lower resolution, lower contrasts and reduced fat saturation (average grade: +1.12) (Fig. 4), the overall image quality was still better on MAVRIC (average grade: −1.88). Quantitatively, the area of metal artifact measured using MAVRIC (range: 43 – 107 cm^2^, average: 61.4 cm^2^) was less compared to STIR (range: 76.3 – 246 cm^2^, average: 135.9 cm^2^). Clinically, MAVRIC was able to detect fluid collection surrounding the implant in one patient. Although the entire body of the patient was swollen and covered with eruption which was suggestive of toxic shock syndrome, thrombotic thrombocytopenia purpura/hemolytic uremic syndrome, or adult stills syndrome, MAVRIC acquisition revealed fluid collection surrounding the endoprosthesis (Fig. 3b), which lead to local puncture and diagnosis of methicillin-sensitive Staphylococcus aureus infection. The patient underwent irrigation and at the final follow-up, the patient is well with no symptom.

## Discussion

There are many histological subtypes of sarcoma of both bone and soft tissues, and treatment is increasingly becoming case-specific with the advent of genetic testing. Several molecular targeting agents have been reported to improve the prognosis and many more are set to be reported. Although, these medical therapies are important, almost all sarcomas need some kind of surgical intervention to eradicate the tumor. It is especially important to perform a wide resection of the lesion to prevent local recurrence from the residual sarcoma cells. To reconstruct large defects, several methods have been proposed including endoprosthesis, allografts, autografts, and recycled bone by pasteurization or liquid nitrogen. When the defect is situated around the joint, endoprosthesis is often utilized due to the poor outcome using other reconstruction methods.

In order to improve the outcome of sarcoma patients, it is important to control the various postoperative adverse events such as acute infection and local recurrence as well as the distant metastasis which is a devastating event both physically and psychologically. Infection leads to multiple salvage operations and delay of chemotherapy which is a poor prognosticator of survival. Local recurrences have been reported to cause higher morbidity due to consequent distant metastasis [9]. One of the reasons for poor outcome after local recurrence is the difficulty of additional surgery. It is difficult to ascertain the tumor infiltration after multiple operations and it is even more difficult after endoprosthesis placement. One of the method recently reported utilize positron emission tomography (PET) - CT to localize the recurrence in the vicinity of metal implants [10]. We have also reported on the possibility of local recurrence detection using PET-CT, where SUV_max_ of 5.0 or greater might differentiate between tumor and fibrosis (data not shown). But, utilization of CT still causes artifacts due to beam hardening which pose significant challenge in delineating the tumor for successful surgery. Novel method for early diagnosis of postoperative adverse event is imperative to improve the outcome of sarcoma patients.

MRI has improved the prognosis of sarcoma patients by accurately detecting the localization and spread of the tumor prior to operation. It has the highest spatial resolution and tissue contrasts compared to other imaging modalities, but metal implants after surgery have impeded its accuracy by causing artifacts that results from metal disturbing the main magnetic field and inducing strong and spatially - varying local gradients [11]. MRI implementation on tumor endoprosthesis including expandable prosthesis has been reported to be safe without local hotness or unintentional lengthening [12]. MAVRIC and the slice encoding for metal artifact attenuation technique has shown promising results by reducing artifacts. MAVRIC has been reported in several reports after orthopaedic implant surgery as a possible solution for suppression of artifacts [4, 6, 7, 13]. MAVRIC has been able to detect joint abnormalities such as joint effusion and bursitis in painful hip [4] and shoulder [7], and showed clinical relevance after total knee replacements [14]. Results from this study also demonstrate that MAVRIC correction for metal induced artifacts improved postoperative visualization around the endoprosthesis. Fortunately, no local recurrence was detected in this case series, but imaging finding of local fluid collection was confirmed by subsequent invasive treatment in one case.

A limitation of this study is its relatively small sample size, which is consistent with other past small case series. Although the preparation of images is still time consuming, it is well suited for dedicated application in areas under suspicion. Further improvement, such as the introduction of MAVRIC - T1WI will enable the use of gadolinium - enhancement and increase the sensitivity for small lesions near the metal implants.

## Conclusion

Several variations of metal artifact suppression technique such as FSE, patient positioning, and use of high bandwidth have been reported [5, 15–17], but there is still significant challenge in detecting small lesions near metal implants. MAVRIC significantly improved the image quality and has the potential to improve patient managements. However, further prospective randomized studies are needed to establish the optimum use of MAVRIC for early diagnosis of local recurrences after sarcoma surgeries.
